# Development and Validation of the Stanford Obstetric Recovery Checklist (STORK): A Delphi Consensus and Multicenter Clinical Validation Study

**DOI:** 10.1001/jamanetworkopen.2025.5713

**Published:** 2025-04-17

**Authors:** Pervez Sultan, Perman Pandal, Anarghya Murthy, Nan Guo, Michaela K. Farber, Paloma Toledo, Nicole Higgins, Julio F. Fiore, Benjamin W. Domingue, Elahe Khorasani, Sally E. Jensen, Deirdre J. Lyell, Brendan Carvalho

**Affiliations:** 1 Department of Anesthesiology, Perioperative and Pain Medicine, Stanford University School of Medicine, Stanford, California; 2 Department of Anesthesiology, Perioperative and Pain Medicine, Brigham and Women’s Hospital, Boston, Massachusetts; 3 Department of Anesthesiology, Miller School of Medicine, Miami, Florida; 4 Department of Anesthesiology, Northwestern University, Chicago, Illinois; 5 Division of General Surgery, McGill University, Montreal, Quebec, Canada; 6 Graduate School of Education, Stanford University, Stanford, California; 7 Department of Obstetrics and Gynecology, Stanford University School of Medicine, Stanford, California

## Abstract

**Background:**

Existing patient-reported outcome measures (PROMs) evaluating outpatient postpartum recovery lack content validity and were mostly not designed for this population. A Delphi process was performed, aiming to develop a patient-reported outcome measure for outpatient postpartum recovery and then evaluate it in a multicenter cohort study.

**Methods:**

Development of the Stanford Obstetric Recovery Checklist (STORK) involved 3 phases: (1) postpartum recovery questions were identified in published reviews; (2) after institutional review board approval, 16 multidisciplinary experts and patient stakeholders participated in 3 Delphi rounds (January 11 to April 12, 2021) to select items, resulting in the development of STORK (47 items; total score range, 0-188, with 0 indicating the worst recovery and 188 indicating the best recovery); and (3) cognitive debriefing interviews were conducted with 10 postpartum individuals to finalize STORK items. Individuals then completed STORK during their inpatient stay and at 2, 6, and 12 weeks post partum in a prospective, 3-center, US longitudinal cohort study conducted from June 13, 2022, to February 28, 2023. Recruitment occurred until 300 six-week STORK surveys were completed. STORK was evaluated at 6 weeks for validity (ability to measure recovery), reliability, and responsiveness. Validity included (1) structural validity (exploratory factor analysis using root mean square residual [RMSR]; <0.08 indicates a good fit); (2) convergent validity (correlation with global health visual analog scale score [GHVAS; scale, 0-100] and EuroQoL Five-Dimensions Three-Levels [EQ-5D-3L]); (3) discriminant validity (mean difference in STORK scores with GHVAS <70 vs ≥70); and (4) confirmatory telephone interviews with postpartum individuals scoring the highest and lowest 10th percentiles of STORK scores. Reliability (consistency of STORK scores) was evaluated using Cronbach α, interitem correlation, split-half reliability, and floor and ceiling effects. Responsiveness (ability of STORK to detect changes in recovery over time) was evaluated using percentage change in score from baseline to 12 weeks.

**Results:**

A total of 525 individuals were recruited after all delivery modes (response rate, 62% [324 of 525] at 6 weeks); 498 (mean [SD] age, 33.3 [4.9] years) completed baseline inpatient postpartum surveys. STORK demonstrated validity: (1) a 4-factor model was the best fit (RMSR = 0.05); (2) correlation with GHVAS scores was ρ = 0.52 (95% CI, 0.43-0.61), and correlation with EQ-5D-3L scores was ρ = −0.67 (95% CI, –0.76 to –0.63); (3) STORK was able to discriminate between patients reporting good and poor recovery (good recovery: median STORK score, 151 [IQR, 136-163] vs poor recovery: median STORK score, 129 [IQR, 107-148]; *P* < .001); and (4) the highest and lowest scores corresponded to subjective assessments. STORK demonstrated reliability (Cronbach α = 0.92; interitem correlation *r* = 0.20; and split-half reliability ρ = 0.98). It also demonstrated responsiveness: percentage increases in overall STORK scores from baseline to week 12 were 19% after spontaneous vaginal delivery, 31% after operative vaginal delivery, 27% after scheduled cesarean delivery, and 20% after nonscheduled cesarean delivery (*P* < .001).

**Conclusion:**

In this cohort study of US individuals, STORK was found to be a valid, reliable, and responsive measure of outpatient postpartum recovery. Future clinical trials are needed to determine its clinical utility.

## Key Points

**Question:**

Can a patient-reported outcome measure be used to assess outpatient postpartum recovery?

**Findings:**

Development of STORK (Stanford Obstetric Recovery Checklist) was achieved after systematic review of the literature and a 3-round Delphi process involving 16 multidisciplinary experts and patient stakeholders; STORK was then evaluated in a multicenter cohort study involving 498 individuals who completed surveys after all delivery modes. At 6 weeks (response rate, 62%), STORK performed well in measures of validity, reliability, and responsiveness.

**Meaning:**

This study suggests that STORK is a valid measure of outpatient postpartum recovery, which can be considered to evaluate individuals in the outpatient setting at 6 weeks post partum.

## Teaser

This study describes the development of a global patient-reported outcome measure for outpatient postpartum recovery and evaluates its validity, reliability, and responsiveness in a US cohort.

## Introduction

Approximately 3.6 million individuals per year in the US and 134 million individuals per year worldwide experience childbirth.^1,2^ Patient-reported outcome measures (PROMs) are inexpensive tools that can be used to efficiently screen the health of large populations and require minimal health care worker training to use.^3^ Although PROMs are considered the criterion standard measures of recovery after childbirth^4^ and have been used to assess individual domains of recovery,^5^ postpartum recovery is a unique and complex construct, making it difficult to define or comprehensively measure.

Inpatient and outpatient postpartum recovery are unique multifactorial constructs.^6^ The 10-item Obstetric Quality of Recovery (ObsQoR-10) PROM is the best currently available measure of inpatient postpartum recovery after all delivery modes and has been validated in multiple health care settings and languages.^7,8,9,10^ Outpatient recovery, however, is a more complex construct, consisting broadly of 13 proposed domains (physical function, anesthesia- and surgery-related complications, pain, sleep, fatigue, motherhood experience, psychosocial distress, psychosocial support, cognition, infant health, feeding and breast health, sexual function, and appearance and cosmetic factors), making comprehensive assessment challenging.^11^ A PROM designed and validated for use at the 6-week obstetric outpatient follow-up clinic visit could be useful for clinicians to facilitate discussion and counseling. A series of published systematic reviews have identified best available PROMs for several outpatient postpartum recovery domains (pain, sleep, fatigue, depression, and anxiety)^12,13,14,15^ and for global (overall) postpartum recovery.^16^ However, existing PROMs of global outpatient postpartum recovery (those that assess >3 recovery domains) have significant limitations.^16^ In particular, they lack content validity, and most were not designed to assess postpartum-specific recovery domains, such as breastfeeding and maternal-neonatal bonding.

The Delphi method has become well established to harness expert opinions surrounding a topic to achieve consensus within an expert group and can be used to develop new condition-specific PROMs, such as those needed to evaluate postpartum recovery. The aim of this study was to first develop a global outpatient postpartum recovery PROM through Delphi consensus with multidisciplinary stakeholders and patient representatives and then to assess its psychometric properties (validity, reliability, and responsiveness) in a geographically diverse US multicenter setting.

## Methods

### PROM Development

After institutional review board approval from Stanford University (ID number 58677), PROM development was undertaken in line with PROMIS (Patient-Reported Outcome Measurement Information System) methods with expert input (S.J.).^17^ Development of the Stanford Obstetric Recovery Checklist (STORK) PROM consisted of 3 phases:

1. Identification of existing questions related to postpartum recovery through a published systematic review and recommendations made by the International Consortium for Health Outcomes Measurement.^16,18^ A list of the 16 PROMs from which items were selected is provided in eTable 1A in Supplement 1.^16^
2. Selection of STORK items through a Delphi approach (January 11 to April 12, 2021). The Delphi process included a panel of 16 expert stakeholders from the fields of obstetrics (n = 1), maternal-fetal medicine (n = 2), postpartum recovery (n = 3), pain (n = 1), epidemiology (n = 1), psychiatry (n = 1), sleep (n = 2), nursing and midwifery (n = 1), and PROM development (n = 1), as well as patient representatives with lived experience of childbirth within the previous 5 years (n = 3). The areas of expertise and race and ethnicity of Delphi participants are provided in eTable 1B in Supplement 1, and the numbers of items considered in each of the 3 Delphi rounds are provided in eTable 1C in Supplement 1. The aim of the Delphi consensus was to develop a new PROM to measure well-being and health status through the assessment of key domains (aspects or dimensions) of postpartum recovery, primarily for use at 6 weeks after childbirth but also for use at different time points after hospital discharge up to 3 months post partum.
3. Cognitive debriefing interviews with 10 postpartum individuals to trial and obtain feedback on the proposed STORK items. eTable 2 in Supplement 1 summarizes the demographic, obstetric, and neonatal variables of individuals undergoing the interviews.

A detailed summary of the Delphi and cognitive debriefing methodology is provided in eMethods 1 in Supplement 1.

### Multicenter STORK Clinical Validation Cohort Study

After institutional review board approval from Stanford University, Northwestern University, and Brigham and Women’s Hospital, individuals were approached and recruited for a cohort study evaluating STORK during their postpartum hospitalization between June 13, 2022, and February 28, 2023. This study follows the Strengthening the Reporting of Observational Studies in Epidemiology (STROBE) reporting guideline for cohort studies.^19^

#### Inclusion and Exclusion Criteria

We included English-speaking women older than 18 years of age who were also able to read and understand written English. All delivery modes were considered, including spontaneous vaginal delivery (with or without neuraxial anesthesia for labor analgesia), operative vaginal delivery (including women experiencing episiotomy or perineal tears and manual removal of placenta), and cesarean delivery (both scheduled and unscheduled). All anesthesia modes were included for individuals undergoing cesarean delivery or labor analgesia, and individuals of all gestations (singleton or twins), gestational ages, and parity were approached to participate. Individuals were excluded if they did not meet the previously described criteria, if they refused to participate, or if they were unable to read or understand written English.

Individuals were approached and recruited within 12 to 36 hours after delivery based on research team availability. Written informed consent was obtained for individuals who participated in this study. Recruitment continued until 100 six-week responses were completed in each of the 3 participating institutions. Consenting individuals were followed up longitudinally to evaluate their quality of postpartum recovery at baseline (day of recruitment) and at 2, 6, and 12 weeks post partum. To maximize PROM completion and response rates, individuals received up to $50 in gift cards on successful completion of all surveys ($5 for completing the week 2 postpartum survey, $30 for completing the week 6 postpartum survey, and $15 for completing the week 12 postpartum survey).

#### Data Collection

Baseline (day of recruitment) data collection included demographic, medical, obstetric, and anesthetic data, as well as level of social support (a lot, some, minimal, and none). At each subsequent time point (weeks 2, 6, and 12 post partum), individuals were invited to complete 2 electronic PROMs using REDCap software (Research Electronic Data Capture, version 14.1.4; Vanderbilt University) after email and text reminders. The first PROM was the newly developed STORK PROM. Each of the 47 STORK items has 5 possible Likert responses scored between 0 and 4 (total possible score ranges, 0-188, with 0 indicating the worst recovery and 188 indicating the best recovery). Self-reported time taken for STORK completion was also collected. The second PROM was the EuroQoL Five-Dimensions Three-Levels (EQ-5D-3L; a validated PROM developed in nonobstetric patients; permission was obtained to use this tool^20^). The EuroQoL also contains a global health visual analog scale (GHVAS) scored between 0 and 100 (where 0 indicates the worst imaginable health state and 100 indicates the best imaginable health state).

#### Outcomes

Psychometric properties of STORK were assessed at 6 weeks post partum (primary outcome time point) using measures of (1) validity and (2) reliability and across all time points using measures of (3) responsiveness and (4) feasibility. Validity (ie, the ability of STORK to measure outpatient postpartum recovery) included several elements:

1. Structural validity: STORK items were evaluated for dimensionality, model fit, item and scale properties, and differential item functioning analysis.
2. Convergent validity: correlation with the EQ-5D-3L score and GHVAS score.
3. Discriminant validity: mean difference in STORK score between individuals reporting a good vs poor recovery (defined by GHVAS score of ≥70 vs <70).
4. Hypothesis testing: we hypothesized that significantly worse STORK scores would be demonstrable at 6 weeks among primiparous vs multiparous individuals; individuals with neonates that required neonatal intensive care (NICU) admission vs no NICU admission; individuals who delivered a neonate with Apgar scores at 5 minutes of less than 7 compared with 7 or more; and individuals who experienced a prolonged hospital stay (>3 days after vaginal delivery or >5 days after cesarean delivery) vs a standard length of stay.
5. Confirmatory telephone interviews were conducted with 20 individuals at Stanford University reporting the lowest (0-10th percentile) and highest (90th-100th percentile) overall STORK scores to evaluate whether research team assessments corresponded to individual poor and good STORK recovery scores, respectively.

Reliability (ie, the consistency of STORK scores) was evaluated using (1) internal consistency (Cronbach α); (2) interitem correlation; (3) split-half reliability; and (4) floor and ceiling effects (ideally, <15% of individuals should achieve the highest or lowest possible PROM score). Responsiveness (ie, the ability of STORK to detect changes in recovery over time) was evaluated by percentage change in global or individual domain scores over time from hospital discharge to week 12 post partum. Feasibility included response rate and median self-reported time to completion.

### Statistical Analysis

The sample size for this study is guided by previous validation studies^6,7,8,21^ and from expert advice (S.J.). We anticipated a dropout or nonresponse rate of approximately 20% (approximately 60 nonresponders) in the outpatient setting. Analyses were performed using Stata, version 14.0 (StataCorp LLC). Data are presented as mean (SD) values, median (IQR) values, and numbers (percentages), with 95% CIs as appropriate. Continuous data were tested for normality using the Shapiro-Wilk normality test, with all percentages rounded up to the nearest integer. Factor validity was evaluated by exploratory factor analysis using root mean square residual (RMSR; <0.08 indicates a good fit). Correlations were calculated using Pearson correlation coefficients for normally distributed data or Spearman correlation coefficients for nonnormally distributed data (ρ = 0.4 to <0.7 considered a moderate correlation and ρ ≥ 0.7 to 1 considered a strong correlation). Continuous data were compared by 1-way analysis of variance for normally distributed data or Wilcoxon signed rank test for nonnormally distributed data. Categorical data were compared by an uncorrected χ^2^ test or Fisher exact test between each delivery mode at each time point. Internal consistency was assessed using Cronbach α and interitem correlation and split-half reliability tests. Responsiveness was assessed using Cohen effect size with a bootstrapped confidence interval. The null hypothesis was rejected if 2-tailed *P* < .05.

## Results

The Delphi process resulted in consideration of 500 items in round 1, 165 items in round 2, and 47 items in round 3. A breakdown of numbers of questions considered in each round by domain is provided in eTable 1C in Supplement 1. After 3 rounds of the Delphi process and cognitive debriefing interviews, the 47-item STORK measure was evaluated among 10 postpartum individuals who delivered at Stanford University. A summary of demographic, obstetric, anesthesia, and neonatal variables for these individuals is provided in eTable 2 in Supplement 1.

A total of 525 individuals were recruited across the 3 centers during the study period. Table 1 provides a summary of demographic, delivery, and recovery data for 498 individuals (mean [SD] age, 33.3 [4.9] years; 6 American Indian or Alaska Native individuals [1%], 71 Asian individuals [14%], 37 Black individuals [7%], 69 Hispanic individuals [14%], 2 Native Hawaiian or Other Pacific Islander individuals [0.4%], 315 White individuals [63%], and 16 individuals of multiple races and ethnicities [3%]) who completed baseline inpatient postpartum surveys. The Figure summarizes the numbers of completed survey responses at each time point across all institutions. The Box provides a content summary of the 47 STORK questions grouped into 4 recovery domains (physical health; mental and emotional health; motherhood experience and social support; sleep and fatigue). The 6-week response rate was 62% (324 of 525) after all delivery modes. eTable 3 in Supplement 1 provides a comparison of demographic, obstetric, and neonatal factors between individuals who responded to surveys (n = 259) and those who were lost to follow-up (n = 266) across all time points.

**Table 1.** Summary of Demographic, Obstetric, Neonatal, and Anesthesia Variables by Delivery Mode

Variable	SVD or induction to SVD (n = 259)	Operative vaginal delivery (n = 17)	CD	
			Scheduled (n = 132)	Nonscheduled (n = 90)
Institution, No. (%)				
Stanford University	57 (22)	4 (24)	40 (30)	6 (7)
Northwestern University	107 (41)	3 (18)	64 (49)	55 (61)
Brigham and Women’s Hospital	95 (37)	10 (58)	28 (21)	29 (32)
Demographics, No. (%)				
Age, mean (SD), y	32.9 (4.6)	32.8 (3.8)	34.5 (5.4)	32.6 (5.0)
Educational level				
High school or below	25 (10)	0	13 (10)	9 (10)
Some college, no degree	16 (6)	0	5 (4)	7 (7)
College degree	111 (43)	7 (41)	58 (44)	25 (28)
Postgraduate degree	79 (31)	9 (53)	45 (34)	38 (42)
Prefer not to respond	28 (10)	1 (6)	11 (8)	11 (12)
Employment status				
Employed or self-employed	182 (70)	15 (88)	91 (69)	68 (76)
Student	5 (2)	1 (6)	1 (1)	2 (2)
Homemaker or not working	43 (17)	0	28 (21)	7 (8)
Prefer not to respond	29 (11)	1 (6)	12 (9)	13 (14)
Ethnicity (Hispanic)	38 (15)	1 (6)	20 (15)	10 (11)
Race				
American Indian or Alaska Native	3 (1)	0	2 (2)	1 (1)
Asian	39 (15)	3 (18)	21 (16)	8 (9)
Black	15 (6)	0	10 (8)	12 (13)
Native Hawaiian or Other Pacific Islander	0	0	1 (1)	1 (1)
White	161 (62)	13 (76)	86 (65)	55 (61)
Multirace	13 (5)	0	1 (1)	2 (2)
Prefer not to respond	28 (11)	1 (6)	11 (8)	11 (12)
Level of social support				
None	110 (42)	4 (24)	65 (49)	55 (61)
Minimal	7 (3)	0	0	1 (1)
Some	28 (11)	0	14 (11)	6 (7)
Lots	114 (44)	13 (76)	53 (40)	28 (31)
Obstetric and complication variables, No. (%)				
Gestational age, median (IQR), wk	39.2 (38.4-39.7)	39.2 (38.0-40.0)	39 (37.4-39.3)	39.4 (38.1-40.0)
Primiparous	119 (46)	14 (82)	39 (30)	69 (77)
Parity, median (IQR)	1 (0-1)	0	1 (0-1)	0
Gravidity, median (IQR)	2 (1-3)	1 (1-1)	2 (1-3)	1 (1-2)
Previous SVD	128 (49)	2 (12)	14 (11)	12 (13)
Previous OVD	12 (5)	1 (6)	3 (2)	1 (1)
Previous elective CD	5 (2)	1 (6)	47 (36)	3 (3)
Previous urgent CD	4 (2)	0	33 (25)	10 (11)
Induction of labor	149 (58)	11 (65)	0	59 (66)
Augmentation of labor				
AROM	131 (51)	9 (53)	0	46 (51)
Oxytocin infusion	169 (65)	10 (59)	0	55 (61)
Maternal age >40 y	6 (2)	0	10 (8)	4 (4)
Oligohydramnios	1 (0.4)	0	2 (2)	2 (2)
Gestational diabetes	19 (7)	3 (18)	10 (8)	7 (8)
Multiple gestation	3 (1)	0	6 (5)	0
Preeclampsia	14 (5)	1 (6)	8 (6)	9 (10)
Chorioamnionitis	5 (2)	0	1 (1)	5 (6)
Other obstetric history^a^	48 (19)	2 (12)	45 (34)	19 (21)
Perineal tear				
First or second degree	80 (31)	4 (24)	0	0
Third or fourth degree	6 (2)	5 (29)	0	0
Estimated blood loss, median (IQR), mL	200 (122-340)	335 (249-400)	700 (490-850)	700 (600-900)
Transfusion requirement^b^	4 (2)	0	3 (2)	6 (7)
Length of hospital stay, median (IQR), h	53.6 (44.8-63.5)	53.1 (49.8-62.2)	76.6 (65.8-92.4)	97.3 (84.3-110.7)
Anesthesia variables				
ASA physical status				
2	221 (85)	16 (94)	108 (82)	68 (76)
3	37 (15)	1 (6)	24 (18)	22 (24)
Labor epidural	145 (56)	14 (82)	NA	35 (39)
Mode of anesthesia (operating room)				
CSE	NA	7 (41)	42 (32)	50 (56)
Spinal	NA	0	90 (68)	12 (13)
Intrapartum epidural	NA	9 (53)	0	24 (27)
General anesthesia	NA	0	0	2 (2)
Unknown	NA	0	0	2 (2)
Epidural blood patch	1 (0.4)	0	1 (1)	1 (1)
Neonatal variables				
NICU admission	13 (5)	0	13 (10)	6 (7)
Step-up neonatal care^c^	58 (22)	4 (24)	33 (25)	6 (7)

Abbreviations: AROM, artificial rupture of membrane; ASA, American Society of Anesthesiologists; CD, cesarean delivery; CSE, combined spinal-epidural; NA, not applicable; NICU, neonatal intensive care unit; OVD, operative vaginal delivery; SVD, spontaneous vaginal delivery.

^a^ Other obstetric history includes breech presentation, gestational hypertension, eclampsia, premature rupture of membranes, preterm premature rupture of membranes, placental abruption, placenta previa, or placenta accreta spectrum disorder.

^b^ Transfusion refers to administration of packed red blood cells, fresh frozen plasma, cryoprecipitate, or platelets.

^c^ Step-up neonatal care is care offered at a higher level than the postpartum ward care but less than NICU-level care.

**Figure.**
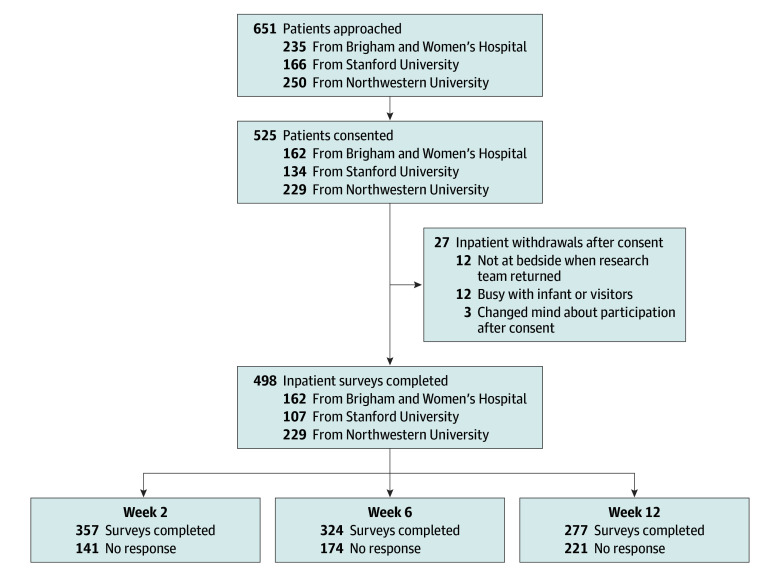
Flowchart of Individuals Who Were Recruited and Numbers of Completed Postpartum Surveys

Box. **Summary of STORK Domains and Question Content Summary**

**Physical health**

- Usual activities
- Satisfaction with usual activities
- Standing from sitting
- Bending, kneeling, or stooping
- Satisfaction with healing after delivery
- Interest in sexual activities
- Physical health
- Difficulty working or performing activities
- Fatigue interfering with physical functioning
- Need for medical treatment
- Pain interference with daily activities
- Pelvic pain
- Breast soreness

**Mental and emotional health**

- Satisfaction with infant health
- Physical appearance
- Aches or pain
- Pain interfering with mood
- Pain interfering with life enjoyment
- Worries
- Helpless
- Anxious
- Worthless
- Hopeless
- Lonely
- Childcare and work conflict
- Memory problems
- Problem-solving
- Life not worth living

**Motherhood experience and social support**

- Access to medical care
- Safety
- Ability to feed infant
- Comfortable with feeding
- Care of infant’s needs
- Trust in instincts of caring for infant
- Relax and enjoy time with infant
- Satisfaction with infant’s health
- Infant growth and development
- Anxiety interfering with mothering ability
- Worry about finances
- Unexpected pregnancy

**Sleep and fatigue**

- Sleep satisfaction
- Rested in morning
- Enough time to rest
- Difficulty falling asleep
- Daytime sleepiness
- Pain interfering with sleep
- Fatigue

Abbreviation: STORK, Stanford Obstetric Recovery Checklist.

### Psychometric Evaluation of STORK

Six-week STORK responses were used to evaluate STORK validity and reliability. STORK demonstrated structural validity. STORK dimensions were best defined with a 4-factor model (RMSR = 0.05; eMethods 2 in Supplement 1); items were evaluated for dimensionality, model fit, item and scale properties, and differential item functioning for key groups (age, educational level, race and ethnicity, literacy; item response theory analysis summary is provided in eTable 4A and B in Supplement 1). Differential item functioning analysis showed that question 6 (interest in sexual activities) and question 16 (aches and pains) demonstrated differences in performance at baseline vs 6 weeks (eTable 4B in Supplement 1). These differences are likely to be due to changes in experiences of these constructs over time. Correlation with GHVAS scores was ρ = 0.52 (95% CI, 0.43-0.61) and with EQ-5D-3L scores was ρ = −0.67 (95% CI, –0.76 to –0.63). STORK was able to discriminate between patients reporting good and poor recovery (good recovery: median STORK score, 151 [IQR, 136-163] vs poor recovery: median STORK score, 129 [IQR, 107-148]; *P* < .001). Findings for convergent validity, discriminant validity, and hypothesis testing for STORK at 6 weeks are summarized in Table 2. Research team interview assessments of individuals with the lowest (0-10th percentile) STORK scores were consistent with patient reports of poor postpartum recovery and with the highest (90th-100th percentile) STORK scores were consistent with patient reports of good postpartum recovery.

**Table 2.** Summary of Psychometric Evaluation of STORK at 6 Weeks Post Partum

Psychometric property and description	Findings	Comments
**Validity**		
Convergent validity		
Correlation of STORK with EQ-5D-3L score	ρ = −0.67 (95% CI, −0.76 to −0.63)	Moderate negative correlation with EQ-5D-3L score (*P* < .001)
Correlation of STORK with GHVAS score	ρ = 0.52 (95% CI, 0.43 to 0.61)	Moderate correlation with GHVAS score (*P* < .001)
Discriminant validity		
Difference in STORK score between patients reporting a good recovery (GHVAS score ≥70) vs poor recovery (GHVAS score <70)	Median score, 151 (IQR, 136 to 163) vs 129 (IQR, 107 to 148)	Mean difference, 22 (*P* < .001)
Hypothesis testing (difference in STORK score per feature)		
Primiparous vs multiparous	Median score, 144 (IQR, 125 to 159) vs 151 (IQR, 136 to 162)	Mean difference, 7 (*P* = .02)
NICU vs no NICU stay	Median score, 134 (IQR, 121 to 151) vs 149 (IQR, 132 to 163)	Mean difference, 29 (*P* = .04)
5-min Apgar score <7 vs ≥7	Median score, 121 (IQR, 104 to 134) vs 149 (IQR, 134 to 162)	Mean difference, 28.5 (*P* = .004)
Prolonged vs not prolonged maternal hospital stay	Median score, 138 (IQR, 123 to 153) vs 149 (IQR, 134 to 162)	Mean difference, 11.5 (*P* = .01)
**Reliability**		
Internal consistency	Cronbach α = 0.92	Good internal consistency
Interitem correlation	0.20	Weak interitem correlation
Split-half reliability	ρ = 0.98	Strong split-half reliability
Floor and ceiling effects	0% Across all time points	Minimal floor or ceiling effects (ie, <15% of patients scoring the lowest or highest possible score)
**Feasibility**		
Response rates	Inpatient, 94% At 2 wk, 68% At 6 wk, 62% At 3 mo, 53%	Response rates decreased with time after childbirth
Completion times	Inpatient, 10 min (IQR, 8 to 10 min); range, 2 to 30 min At 2 wk, 7 min (IQR, 5 to 10 min); range, 2 to 30 min At 6 wk, 5 min (IQR, 5 to 10 min); range, 2 to 60 min At 12 wk, 5 min (IQR, 5 to 10 min); range, 1 to 30 min	Completion time as self-reported by the patient

Abbreviations: EQ-5D-3L, EuroQoL Five-Dimensions Three-Levels measure; GHVAS, global health visual analog scale; NICU, neonatal intensive care unit; STORK, Stanford Obstetric Recovery Checklist.

STORK demonstrated reliability (Cronbach α = 0.92; interitem correlation *r* = 0.20; and split-half reliability ρ = 0.98) (Table 2). It also demonstrated responsiveness: STORK scores increased over the 12-week period. Percentage increases in overall STORK scores from baseline to week 12 were 19% after spontaneous vaginal delivery, 31% after operative vaginal delivery, 27% after scheduled cesarean delivery, and 20% after nonscheduled cesarean delivery (*P* < .001). STORK was responsive up to 12 weeks post partum; the Cohen *d* effect size and standardized response means of STORK score change over time per delivery mode are summarized in eTable 5 in Supplement 1. Measures of feasibility are summarized in Table 2. The median completion times as reported by individuals were between 5 and 10 minutes over the study period.

## Discussion

In this cohort study of postpartum individuals delivering across 3 US states, the STORK measure performed well in measures of validity, reliability, responsiveness, and feasibility up to 3 months post partum. The rigorous development with content experts and validation of this measure across a diverse geographical US patient cohort supports further study of this measure as a tool to evaluate US individuals as part of their 6-week obstetric follow-up visit.

PROMs are easy to use and low in cost, which support their use for screening large numbers of patients. A previous review evaluating validated PROMs for outpatient postpartum recovery concluded that existing measures were not robustly developed or validated, highlighting the need for a new PROM of outpatient postpartum recovery.^16^ STORK addresses the issues identified in the previous review through the robust methods used in its development and validation, which are consistent with recommendations from PROMIS, the US Food and Drug Administration,^22^ and COSMIN (Consensus‐Based Standards for the Selection of Health Measurement Instruments) initiatives.^23^

The Postpartum Assessment of Health Survey (PAHS) is a measure developed for the assessment of patients as a 12- to 14-month follow-up to the Centers for Disease Control and Prevention’s Pregnancy Risk Assessment Monitoring System (PRAMS).^24,25^ The PAHS consists of more than 110 questions (many items contain additional stems) and takes approximately 60 minutes to complete. The PAHS does not ask questions about postpartum sleep, motherhood experience (adapting to maternal role and maternal-neonatal bonding), infant health, sexual function, cognition, and appearance and cosmetic factors, which are important aspects of postpartum recovery.^11^ Similarly, the American College of Obstetricians and Gynecologists postpartum care checklist proposes several domains for the individual to talk about with their obstetrician.^26^ However, this checklist is not a PROM and does not result in a score that can be used to track recovery longitudinally. The decision was made by the study group to assess STORK primarily at the 6-week postpartum time point to align with current clinical practice in the US for timing of the postpartum clinic visit. The median time for completion at the 6-week time point was 5 minutes, which supports feasibility for use at this time point. The time taken to complete a PROM must balance a comprehensive evaluation associated with more questions against the increased likelihood of nonresponse. Further research is needed to evaluate the clinical utility of STORK in the setting of obstetric follow-up clinics and to explore its use as an adjunct to physician consultations. Because the STORK measure demonstrates responsiveness (ie, score improves after childbirth), its role in tracking recovery longitudinally across time points, similar to serial measurements of height or weight of the infant, also warrants further study. The findings from this study show promise for STORK as a measure to potentially profile different domains of postpartum recovery and to identify the domains of recovery associated with lower STORK scores at different time points. The role of STORK in counseling individuals, managing expectations, and comparing recovery trajectories after hospital discharge for individuals with different socioeconomic and demographic variables and across various maternal and neonatal comorbid conditions also requires further study.

STORK has been developed primarily for clinical use but also demonstrates potential for use as a research tool to track response to hospital-instituted interventions that are positively or negatively associated with outpatient recovery. Evaluation in the primary care setting as a screening tool and to assess its role in studies designed to evaluate response to interventions administered after hospital discharge is still required.

This study has performed a psychometric evaluation of STORK in line with tests recommended by the COSMIN collaborative. It demonstrates good structural validity at 6 weeks; however, further studies are needed to evaluate how this measure performs in different hospital settings (eg, rural hospital or private practice care structures), in underrepresented racial and ethnic cohorts, and across different obstetric and medical morbidity groups using differential item functioning analyses and development of translated versions. Furthermore, recovery is a dynamic process, which suggests that certain questions are likely to be more applicable at different time points, such as worry about returning to work, which may be less relevant at week 2 compared with week 12 post partum. Similarly, issues with breastfeeding and pain may be less relevant at month 3 compared with week 2 post partum. STORK may benefit from amended versions that are designed for specific time points. However, use of different amended versions would preclude longitudinal comparisons from the same patient. The overall STORK score provides an easy-to-understand metric in its current form. The testing of this PROM in different forms (eg, paper form vs REDCap vs questions read out by a nurse) also warrants further study. A computer adaptive test may also improve the efficiency of the instrument by omitting questions that are irrelevant to certain individuals. Studies are also needed to identify techniques for optimizing response rates through incentives or use as part of an online check-in process before a consultation.

### Limitations

This study has some limitations. Although the development of this measure was robust with a broad mix of stakeholders, we opted to include questions from only 16 global PROMs rather than the 201 single-domain PROMs identified in a previous scoping review.^5,16^ This choice to include questions from 16 PROMs was to ensure feasibility, maximize compliance, and facilitate timely completion of the 3 rounds of the Delphi process. Stakeholders were also given the opportunity to propose additional questions as part of the Delphi process. We selected an expert panel of multidisciplinary practitioners and physicians based in the US, as we felt that the primary use of this measure would be in this setting. However, we acknowledge that by excluding non-US stakeholders, we may have failed to include items that could be pertinent to recovery in non-US health care settings.

Response rates were good (62%) at 6 weeks. Nonresponders differed from responders in terms of their demographic characteristics (including employment status, highest level of education, and racial and ethnic group) and obstetric, anesthesia, and neonatal variables. Such factors are known to be associated with disparities in maternal health care and outcomes.^27^ Future studies are needed to explore strategies to optimize attendance at the 6-week obstetric follow-up visit and maximize compliance with available maternal health screening programs. We felt it was important to recruit individuals to complete STORK in more than one center to demonstrate generalizability of the tool. However, we acknowledge that individuals were recruited from only 3 academic US centers, individuals were mostly White with a higher median age compared with the national average, and a low number of individuals underwent operative vaginal delivery. Further studies are therefore needed to evaluate STORK in larger numbers of members of racial and ethnic minority groups, in rural and private practice settings, and in other US states, other countries, and in other health care systems with individuals with varying insurance coverage. Furthermore, to improve generalizability of this PROM, individuals who do not speak English should be invited to complete translated versions to assess whether the PROM remains valid, reliable, and responsive. We plan to translate and validate this measure internationally to evaluate its performance in racially, ethnically, and culturally diverse postpartum populations.

## Conclusions

After the development of STORK, this cohort study found it to be a valid, reliable, and responsive PROM of outpatient global postpartum recovery. Future studies are needed to evaluate the clinical utility of STORK by comparing it with standard care for evaluation of outpatient postpartum recovery and to evaluate its performance in different patient groups, languages, and health care settings.

## Notes

Supplement 1. **eTable 1A.** Patient-Reported Outcome Measures Assessing >3 Domains of Postpartum Recovery From Which Items Were Evaluated and Selected for Inclusion in the Delphi Study **eReferences 1**. **eTable 1B.** Summary of Delphi Stakeholders and Area of Expertise **eTable 1C.** Summary of Number of Questions Considered in Each Delphi Round Grouped According to Postpartum Recovery Domains **eTable 2.** Summary of Demographic, Obstetric, Neonatal and Anesthesia Variables of Patients Undergoing Cognitive Debriefing Interviews (n=10) **eTable 3.** Table Comparing Demographic, Obstetric, Neonatal and Anesthesia Variables Between Patients Responding to Surveys and Those Lost to Follow-up **eTable 4A.** Summary of STORK Domains and Question Content Summary **eTable 4B.** Tests of Differential Item Functioning (DIF) Statistics **eTable 5.** Standardized Response Means of STORK **eMethods 1.** Summary of STORK Development **eReferences 2**. **eMethods 2.** Exploratory Factor Analysis of the STORK Questionnaire **eReferences 3**.

Supplement 2. **Nonauthor Collaborators.** Stanford Obstetric Recovery Checklist (STORK) Investigators

Supplement 3.
Data Sharing Statement
