# Supplementary material for: Development and Validation of the Stanford Obstetric Recovery Checklist (STORK): A Delphi Consensus and Multicenter Clinical Validation Study
Source: JAMA Netw Open. 2025 Apr 17;8(4):e255713. doi: 10.1001/jamanetworkopen.2025.5713 (PMC12006865; doi:10.1001/jamanetworkopen.2025.5713)
Supplement: Supplement 1. — eTable 1A. Patient-Reported Outcome Measures Assessing >3 Domains of Postpartum Recovery From Which Items Were Evaluated and Selected for Inclusion in the Delphi Study eReferences 1. eTable 1B. Summary of Delphi Stakeholders and Area of Expertise eTable 1C. Summary of Number of Questions Considered in Each Delphi Round Grouped According to Postpartum Recovery Domains eTable 2. Summary of Demographic, Obstetric, Neonatal and Anesthesia Variables of Patients Undergoing Cognitive Debriefing Interviews (n=10) eTable 3. Table Comparing Demographic, Obstetric, Neonatal and Anesthesia Variables Between Patients Responding to Surveys and Those Lost to Follow-up eTable 4A. Summary of STORK Domains and Question Content Summary eTable 4B. Tests of Differential Item Functioning (DIF) Statistics eTable 5. Standardized Response Means of STORK eMethods 1. Summary of STORK Development eReferences 2. eMethods 2. Exploratory Factor Analysis of the STORK Questionnaire eReferences 3. [file jamanetwopen-e255713-s001.pdf]

Supplementary Online Content

Sultan P, Pandal P, Murthy A, et al; Stanford Obstetric Recovery Checklist (STORK) Investigators. Development and validation of the Stanford Obstetric Recovery Checklist (STORK): a Delphi consensus and multicenter clinical validation study. *JAMA Netw Open.* 2025;8(4):e255713. doi:10.1001/jamanetworkopen.2025.5713

eTable 1A. Patient-Reported Outcome Measures Assessing >3 Domains of Postpartum Recovery From Which Items Were Evaluated and Selected for Inclusion in the Delphi Study
eReferences 1.
eTable 1B. Summary of Delphi Stakeholders and Area of Expertise
eTable 1C. Summary of Number of Questions Considered in Each Delphi Round Grouped According to Postpartum Recovery Domains
eTable 2. Summary of Demographic, Obstetric, Neonatal and Anesthesia Variables of Patients Undergoing Cognitive Debriefing Interviews (n=10)
eTable 3. Table Comparing Demographic, Obstetric, Neonatal and Anesthesia Variables Between Patients Responding to Surveys and Those Lost to Follow-up
eTable 4A. Summary of STORK Domains and Question Content Summary
eTable 4B. Tests of Differential Item Functioning (DIF) Statistics
eTable 5. Standardized Response Means of STORK
eMethods 1. Summary of STORK Development
eReferences 2.
eMethods 2. Exploratory Factor Analysis of the STORK Questionnaire
eReferences 3.

This supplementary material has been provided by the authors to give readers additional information about their work.

© 2025 Sultan P et al. *JAMA Network Open.*

eTable 1A. Patient-Reported Outcome Measures Assessing >3 Domains of Postpartum Recovery From Which Items Were Evaluated and Selected for Inclusion in the Delphi Study

Obstetric specific	Non-obstetric specific
Barkin index of maternal functioning[1]	SF12[9]
Inventory of functional status after childbirth[2]	EQ5D[10]
Maternal concerns questionnaire[3]	WHOQoL-BREF[11]
Maternal postpartum QoL tool[4]	WHO-DAS II[12]
Postpartum symptom checklist[5]	Swedish health related QoL[13]
Rural postpartum QoL[6]	Nottingham health profile[14]
Postpartum QoL[7]	QoL Inventory[15]
SF36[8]	PROMIS-57[16]

Obstetric specific PROMs were specifically developed and validated for use in the postpartum population. References for studies which evaluated these PROMs in the postpartum setting are provided below. An exhaustive list of postpartum validation studies for these measures can be found in the supplement of following article: Sultan P, Sharawi N, Blake L, Ando K, Sultan E, Aghaeepour N, Carvalho B, Sadana N. Use of Patient-Reported Outcome Measures to Assess Outpatient Postpartum Recovery: A Systematic Review. JAMA Netw Open. 2021 May 3;4(5):e2111600. doi: 10.1001/jamanetworkopen.2021.11600. PMID: 34042993

© 2025 Sultan P et al. *JAMA Network Open.*

eReferences 1.

1. Barkin J, Wisner K, Bromberger J, *et al.* Development of the Barkin Index of Maternal Functioning. *J Women's Heal* 2010;**19**:2239–46.
2. Fawcett J, Tulman L, Myers S. Development of the inventory of functional status after childbirth. *J Nurse Midwifery* 1988;**33**:252–60.
3. Sheil E, Bull M, Moxon B, *et al.* Concerns of Childbearing Women: A Maternal Concerns Questionnaire as an Assessment Tool. *J Obstet Gynecol Neonatal Nurs* 1995;**24**:149–55.
4. Hill P, Aldag J, Hekel B, *et al.* Maternal Postpartum Quality of Life Questionnaire. *J Nurs Meas* 2006;**14**:205–20.
5. Maloni J, Park S. Postpartum Symptoms After Antepartum Bed Rest. *J Obstet Gynecol Neonatal Nurs* 2005;**34**:163–71.
6. Huang K, Tao F, Liu L, *et al.* Does delivery mode affect women's postpartum quality of life in rural China? *Midwifery Perinat Care* 2012;**21**:1534–43.
7. Nikan F, Jafarabadi M, Mohammad-Alizadeh-Charandabi S, *et al.* Psychometric Properties of the Iranian Version of a Postpartum Women's Quality of Life Questionnaire (PQOL): A Methodological Study. *Iran Red Crescent Med J* 2016;**18**:e35460.
8. Lydon-Rochelle M, Holt V, Martin D. Delivery method and self-reported postpartum general health status among primiparous women. *Paediatr Perinat Epidemiol* 2001;**15**:232–40.
9. Handa V, Zyczynski H, Burgio K, *et al.* The impact of fecal and urinary incontinence on quality of life 6 months after childbirth. *Am J Obs Gynecol* 2007;**197**:636.e6.
10. Petrou S, Kim S, McParland P, *et al.* Mode of Delivery and Long-Term Health-Related Quality-of-Life Outcomes: AProspective Population-Based Study. *Birth* 2017;**44**:110–9.
11. Webster J, Nicholas C, Velacott C, *et al.* Validation of the WHOQOL-BREF among women following childbirth. *Aust New Zeal J Obstet Gynaecol* 2010;**50**:132–7.
12. Senturk V, Hanlon C, Medhin G, *et al.* Impact of perinatal somatic and common mental disorder symptoms on functioning in Ethiopian women: The P-MaMiE population-based cohort study. *J Affect Disord* 2012;**136**:340–9.
13. Carlander A, Andolf E, Edman G, *et al.* Health-related quality of life five years after birth of the first child. *Sex Reprod Healthc* 2015;**6**:101–7.
14. Baghirzada L, Downey K, Macarthur A. Assessment of quality of life indicators in the postpartum period. *Int J Obstet Anesth* 2013;**22**:209–16.
15. Nieminen K, Berg I, Frankenstein K, *et al.* Internet-provided cognitive behaviour therapyof posttraumatic stress symptoms followingchildbirth - a randomized controlled trial. *Cogn Behav Ther* 2016;**45**:287–306.
16. Cella D; Weinfurt K; Revicki D; Pilkonis P; DeWalt D; DeVellis R; Cook K; Buysse D; Amtmann D; Yount S; Reeve B; Riley W; Stone A; Rothrock NE; Bode R; Choi S; Fries JF; Gershon R; Hahn EA; Lai JS; Rose M; Hays RD. Patient-reported outcome measurement information system – 57 Profile v2.1 (PROMIS-57 Profile v2.1). Available at URL: https://eprovide.mapi-trust.org/instruments/patient-reported-outcomes-measurement-information-system-57-profile-v2.1. Accessed on 22nd Decemeber, 2024.

© 2025 Sultan P et al. *JAMA Network Open.*

eTable 1B. Summary of Delphi Stakeholders and Area of Expertise

Name	Expertise	Gender	Race	Ethnicity
Sultan P	Recovery / PROM development	Male	Asian	Non-Hispanic
Carvalho B	Recovery	Male	White	Non-Hispanic
Sharawi N	Recovery	Male	Black	Non-Hispanic
Jensen S	PROM development	Female	White	Non-Hispanic
Lyell DJ	MFM	Female	White	Non-Hispanic
Brookfield K	MFM	Female	White	Non-Hispanic
Whittington J	Obstetrics	Female	White	Non-Hispanic
Carmichael S	Epidemiology	Female	White	Non-Hispanic
Coker J	Psychiatry	Female	White	Non-Hispanic
Flood P	Postpartum pain	Female	White	Non-Hispanic
Kawai M	Sleep medicine	Male	Asian	Non-Hispanic
Barwick F	Sleep medicine	Female	White	Non-Hispanic
Moreno C	Director of nursing and midwifery	Female	White	Non-Hispanic
Yun R	Patient representative	Female	Asian	Non-Hispanic
Sultan E	Patient representative	Female	Asian	Non-Hispanic
Ansari J	Patient representative	Female	White	Non-Hispanic

PROM=patient-reported outcome measure; MFM=maternal fetal medicine
All experts have published in the field of postpartum recovery in relation to their respective area of clinical interest. Collectively, patient representatives have lived experiences of each delivery mode (cesarean delivery, operative vaginal delivery and spontaneous vaginal delivery)

© 2025 Sultan P et al. *JAMA Network Open.*

eTable 1C. Summary of Number of Questions Considered in Each Delphi Round Grouped According to Postpartum Recovery Domains

Domain	Round 1	Round 2	Round 3
Global health state	20	7	2
Physical function	99	19	5
Surgical / medical factors	27	8	2
Pain	30	14	5
Psychosocial distress	78	28	8
Psychosocial support	110	23	4
Sleep	28	9	4
Fatigue	26	15	2
Motherhood experience	33	15	3
Infant health	7	5	2
Feeding & breast health	10	5	3
Sexual function	15	6	2
Cognition	11	8	3
Appearance & cosmetic factors	6	3	2
Total no. of items	500	165	47

Group discussion and finalization of item and response wording was conducted in Round 3.

© 2025 Sultan P et al. *JAMA Network Open.*

eTable 2. Summary of Demographic, Obstetric, Neonatal and Anesthesia Variables of Patients Undergoing Cognitive Debriefing Interviews (n=10)

Variables	Patients (n=10)
**Demographics**	
Age (yrs)	34±4.3
Ethnicity (Hispanic)	1 (10%)
Race	
White	6 (60%)
Black	0
American Indian/Native	0
Asian	3 (30%)
Native Hawaiian / Other Pacific Islander	0
Multi-race	1 (10%)
**Obstetric variables**	
Gestational age (weeks)	39.7±1.0
Primiparous	4 (40%)
Parity	1 [0-1]
Gravidity	1 [1-2]
Induction of labor	5 (50%)
Augmentation of labor	
AROM	1 (10%)
Oxytocin infusion	5 (50%)
Delivery method	
Spontaneous vaginal delivery	4 (40%)
Caesarean delivery	6 (60%)
Maternal age > 40 years	1 (10%)
Oligohydramnios	0
Gestational diabetes	0
Multiple gestation	0
Preeclampsia	1 (10%)
Chorioamnionitis	0
Perineal tear	
1st / 2nd degree	1 (10%)
3rd / 4th degree	1 (10%)
EBL (mls)	500 [300-1000]
**Anesthesia variables**	
ASA physical status	
2	7 (70%)
3	3 (30%)
**Neonatal variables**	
Apgar score @ 1 min	8 [7-8]
Apgar score @ 5 min	9 [9-9]

Values expressed as mean ± SD or median [interquartile range] unless stated otherwise

© 2025 Sultan P et al. *JAMA Network Open.*

eTable 3. Table Comparing Demographic, Obstetric, Neonatal and Anesthesia Variables Between Patients Responding to Surveys and Those Lost to Follow-up

	**Responders (n=259)**	**Lost to follow- up (n=266)**	**p-value**
**Demographics**			
Age	33.9±4.5	32.7±5.3	0.006
Education			<0.001
High school or below	12 (5%)	39 (15%)	
Some college, no degree	11 (4%)	18 (7%)	
College degree	132 (51%)	82 (31%)	
Postgraduate degree	96 (37%)	82 (31%)	
Prefer not to respond	8 (3%)	45 (17%)	
Current employment			<0.01
Employed/ Self-employed	202 (78%)	172 (65%)	
Student	5 (2%)	5 (2%)	
Homemaker/Not working	44 (17%)	40 (15%)	
Unknown	8 (3%)	49 (18%)	
Ethnicity (Hispanic)	30 (11.6%)	44 (16.5%)	0.10
Race			<0.01
White	166 (64%)	158 (59%)	
Black	15 (6%)	22 (8%)	
American Indian/Native	4 (2%)	3 (1%)	
Asian	55 (21%)	30 (11%)	
Native Hawaiian / Other Pacific Islander	0	2 (1%)	
Multi-race	11 (4%)	6 (2%)	
Unknown	8 (3%)	45 (17%)	
Level of social support			<0.01
None	88 (34%)	148 (56%)	
Minimal	7 (3%)	1 (0.4%)	
Some	31 (12%)	22 (8%)	
Lots	133 (51%)	95 (36%)	
**Obstetric and complication variables**			
Gestational age (weeks)	39.1 [38.0- 39.6]	39.2 [38.3- 39.5]	0.54
Primiparous	128 (49.4%)	113 (42.5%)	<0.01
Parity	1 [0-1]	1 [0-1]	0.52
Gravidity	2 [1-3]	2 [1-3]	0.26
Delivery method			<0.01
Spontaneously vaginal delivery	135 (52%)	124 (47%)	
Operative vaginal delivery	13 (5%)	4 (2%)	
Scheduled C-section	70 (27%)	62 (23%)	
Non-scheduled C-section	41 (16%)	49 (18%)	
Missing	0	27 (10%)	
Previous SVD	77 (30%)	79 (30%)	0.99
Previous OVD	7 (3%)	10 (4%)	0.49
Previous elective CD	31 (12%)	25 (9%)	0.34
Previous urgent CD	24 (9%)	23 (9%)	0.80
Induction of labor	129 (50%)	90 (34%)	<0.01
Augmentation of labor			

© 2025 Sultan P et al. *JAMA Network Open.*

AROM<br>Oxytocin infusion	97 (38%)<br>131 (51%)	89 (34%)<br>103 (39%)	0.34<br>0.01
Maternal age > 40	8 (3%)	12 (5%)	0.40
Oligohydramnios	3 (1%)	2 (1%)	0.63
Gestational diabetes	26 (10%)	13 (5%)	0.02
Multiple gestation	3 (1%)	6 (2%)	0.33
Preeclampsia	20 (8%)	12 (5%)	0.12
Chorioamnionitis	5 (2%)	6 (2%)	0.80
Other obstetric history	62 (24%)	52 (20%)	0.22
Perineal tear<br>1st / 2nd degree<br>3rd / 4th degree	 48 (19%)<br>9 (4%)	 37 (14%)<br>2 (1%)	 0.15<br>0.03
EBL (mls)	457.5 [200-800]	568.5 [250-750]	0.35
Transfusion requirement	10 (4%)	3 (1%)	<0.01
Length of hospital stay (h)	63.4 [50.2-80.5]	65.5 [52.9-85.2]	0.25
**Anesthesia variables**			
ASA physical status			<0.01
2	215 (83.0%)	198 (74.4%)	
3	43 (16.6%)	41 (15.4%)	
Labor epidural n (%)	158 (61.0%)	88 (33.1%)	<0.01
Mode of anesthesia (OR)<br>CSE<br>Spinal<br>Intrapartum epidural<br>General anesthesia<br>Unknown	 58 (23%)<br>44 (17%)<br>18 (7%)<br>1 (0.4%)<br>2 (1%)	 41 (15%)<br>57 (21%)<br>15 (6%)<br>1 (0.4%)<br>28 (11%)	<0.01
Epidural blood patch	3 (1%)	0	0.08
**Neonatal variables**			
NICU	15 (6%)	17 (6%)	<0.01
Step-up neonatal care	89 (34%)	12 (5%)	<0.01

Variables presented as mean ± standard deviation, median [interquartile range] and number (percentage)
SVD=spontaneous vaginal delivery; OVD=operative vaginal delivery; CD=cesarean delivery; EBL=estimated blood loss; other obstetric history includes breech presentation, gestational hypertension, eclampsia, premature rupture of membranes, preterm premature rupture of membranes, placental abruption, placenta previa, placenta accreta spectrum disorder; transfusion refers to administration of packed red blood cells, fresh frozen plasma, cryoprecipitate or platelets; OR=operating room; NICU=neonatal intensive care; step-up ward care is care offered at higher level than postpartum ward care but less than NICU level care; ASA=American Society of Anesthesiologists; CSE=combined spinal-epidural; AROM=artificial rupture of membranes; SD=standard deviation; IQR=interquartile range; 'other race' was selected if a patient did not identify with the presented race options.

© 2025 Sultan P et al. *JAMA Network Open.*

IRT / Differential item functioning analysis

eTable 4A. Summary of STORK Domains and Question Content Summary

Physical Health		Motherhood Experience & Social Support	
1	Usual activities	29	Access to medical care
2	Satisfaction with usual activities	30	Safety
3	Standing from sitting	31	Ability to feed infant
4	Bending, kneeling or stooping	32	Comfortable with feeding
5	Satisfaction with healing after delivery	33	Care of infant's needs
6	Interest in sexual activities	34	Trust in instincts of caring for infant
7	Physical health	35	Relax and enjoy time with infant
8	Difficulty working or performing activities	36	Satisfaction with infant's health
9	Fatigue interfering with physical functioning	37	Infant growth and development
10	Need for medical treatment	38	Anxiety interfering with mothering ability
11	Pain interference with daily activities	39	Worry about finances
12	Pelvic pain	40	Unexpected pregnancy
13	Breast soreness		
**Mental & Emotional Health**		**Sleep & Fatigue**	
14	Satisfaction with infant health	41	Sleep satisfaction
15	Physical appearance	42	Rested in morning
16	Aches or pain	43	Enough time to rest
17	Pain interfering with mood	44	Difficulty falling asleep
18	Pain interfering with life enjoyment	45	Daytime sleepiness
19	Worries	46	Pain interfering with sleep
20	Helpless	47	Fatigue
21	Anxious		
22	Worthless		
23	Hopeless		
24	Lonely		
25	Childcare and work conflict		
26	Memory problems		
27	Problem-solving		
28	Life not worth living		

© 2025 Sultan P et al. *JAMA Network Open.*

eTable 4B. Tests of Differential Item Functioning (DIF) Statistics

	Baseline vs Week 6	Week 2 vs Week 6	Week 12 vs week 6
**Physical Health**			
q1	-0.01	-0.004	-0.005
q2	0.02	0.02	-0.04
q3	-0.008	0.005	-0.003
q4	-0.04	-0.01	-0.02
q5	0.02	0.02	0.01
q6*	0.13	0.05	-0.06
q7	0.07	0.05	-0.06
q8	0.04	0.03	0.02
q9	0.04	-0.005	-0.019
q10	0.003	0.006	0.002
q11	-0.008	-0.01	0.01
q12	-0.02	0.007	-0.02
q13	0.05	-0.06	0.04
**Mental & Emotional Health**			
q14	-0.009	-0.008	-0.01
q15	0.06	0.05	0.01
q16*	-0.14	-0.03	-0.01
q17	-0.03	-0.01	0.02
q18	-0.04	-0.01	0.02
q19	0.006	0.004	-0.0004
q20	0.006	-0.010	0.007
q21	0.03	-0.002	-0.047
q22	0.002	-0.001	0.007
q23	0.005	-0.008	-0.007
q24	0.04	0.02	0.02
q25	0.006	0.02	-0.03
q26	0.03	0.01	-0.01
q27	0.01	0.001	-0.005
q28	0	0	0
**Motherhood Experience & Social Support**			
q29	0.02	0.02	0.02
q30	0.01	0.01	0.007
q31	-0.04	-0.008	-0.002
q32	-0.05	-0.03	-0.006
q33	-0.02	-0.002	0.003
q34	-0.002	0.002	-0.003
q35	-0.003	-0.001	-0.01
q36	-0.01	-0.005	-0.01
q37	0.003	0.02	0.009
q38	0.008	-0.002	-0.006
q39	0.05	0.001	-0.01
q40	-0.003	-0.003	0
**Sleep & Fatigue**			
q41	0.06	-0.02	0.03

© 2025 Sultan P et al. *JAMA Network Open.*

q42	0.06	0.002	0.004
q43	0.03	-0.02	-0.08
q44	-0.07	0.03	-0.05
q45	0.03	0.02	0.03
q46	-0.07	-0.01	0.003
q47	0.01	-0.03	0.03

- The above table summarizes the P-DIF statistic (defined as a weighted average of the difference in proportions of success in the reference group week 6 and other groups).

- Standardized P-DIF is further described at:

  URL: https://rdrr.io/cran/difR/man/difStd.html

- Analysis (unless otherwise indicated) was conducted on complete response strings at the week 6 time point (n=281).

- Numbers bigger than 0.1 are marked with * in Table 2. This indicates potential DIF.

- Overall extensive DIF related to postpartum time was not demonstrable in this data set.

© 2025 Sultan P et al. *JAMA Network Open.*

eTable 5. Standardized Response Means of STORK

	SVD/induction	OVD	Scheduled CD	Non-scheduled CD	Total
	Standardized response means (95% CI)	Standardized response means (95% CI)	Standardized response means (95% CI)	Standardized response means (95% CI)	Standardized response means (95% CI)
**Overall STORK score**					
Week 2 vs baseline	0.63 (0.49, 0.80)	0.45 (-0.06, 1.56)	0.71 (0.45, 1.03)	0.92 (0.55, 1.44)	0.68 (0.56, 0.83)
Week 6 vs baseline	0.80 (0.61, 1.04)	1.40 (0.83, 2.72)	1.28 (0.99, 1.64)	1.32 (1.08, 1.75)	1.00 (0.85, 1.18)
Week 12 vs baseline	1.20 (0.99, 1.45)	1.44 (0.92, 2.63)	1.35 (1.12, 1.72)	1.40 (0.88, 2.25)	1.28 (1.12, 1.47)
**Physical health**					
Week 2 vs baseline	0.93 (0.78,1.12)	0.49 (-0.02, 1.35)	0.68 (0.29, 0.71)	1.01 (0.69, 1.53)	0.83 (0.69, 0.97)
Week 6 vs baseline	1.22 (1.02, 1.48)	1.57 (1.12, 2.63)	1.52 (1.16, 2.04)	1.77 (1.32, 2.55)	1.37 (1.21, 1.56)
Week 12 vs baseline	1.61 (1.37, 1.92)	2.18 (1.94, 2.96)	1.75 (1.51, 2.08)	1.80 (1.11, 3.07)	1.68 (1.48, 1.91)
**Mental & emotional health**					
Week 2 vs baseline	0.15 (-0.01, 0.31)	-0.11 (-0.77, 0.43)	0.32 (0.11, 0.58)	0.19 (-0.10, 0.59)	0.20 (0.09, 0.32)
Week 6 vs baseline	0.15 (-0.01, 0.32)	0.59 (0.14, 1.22)	0.48 (0.27, 0.71)	0.53 (0.26, 0.86)	0.30 (0.19, 0.43)
Week 12 vs baseline	0.32 (0.15, 0.51)	0.45 (-0.05, 1.29)	0.46 (0.25, 0.75)	0.50 (0.17, 0.96)	0.40 (0.27, 0.53)
**Motherhood experience & social support**					
Week 2 vs baseline	0.06 (-0.11, 0.21)	0.36 (-0.19, 1.06)	0.19 (-0.02, 0.40)	0.22 (-0.14, 0.54)	0.14 (0.03, 0.25)
Week 6 vs baseline	0.27 (0.12, 0.42)	0.85 (0.29, 2.24)	0.33 (0.11, 0.53)	0.32 (0.03, 0.65)	0.32 (0.21, 0.43)
Week 12 vs baseline	0.38 (0.22, 0.54)	0.82 (0.25, 2.42)	0.37 (0.15, 0.62)	0.26 (-0.05, 0.63)	0.38 (0.26, 0.51)
**Sleep & fatigue**					
Week 2 vs baseline	0.39 (0.24, 0.55)	0.25 (-0.28, 0.82)	0.59 (0.40, 0.80)	0.72 (0.44, 1.13)	0.49 (0.37, 0.59)
Week 6 vs baseline	0.56 (0.40, 0.73)	0.55 (0.09, 1.14)	0.83 (0.59, 1.11)	0.80 (0.52, 1.25)	0.66 (0.54, 0.79)
Week 12 vs baseline	0.88 (0.69, 1.09)	0.77 (0.25, 1.65)	1.04 (0.75, 1.46)	0.95 (0.62, 1.37)	0.93 (0.78, 1.11)

SVD=spontaneous vaginal delivery; OVD=operative vaginal delivery; CD=cesarean delivery

© 2025 Sultan P et al. *JAMA Network Open.*

eMethods 1. Summary of STORK Development

Phase 1. Identify existing items.

We generated a comprehensive list of PROM items evaluating outpatient recovery domains following all delivery modes by combining all items from existing validated PROMs identified in a published systematic review and as recommended by the International Consortium for Health Outcomes Measurement.[1,2] A full list of the 16 PROMs from which items were selected is provided in eTable 1a.[1]

Phase 2. Delphi consensus surrounding items to include in STORK.

The Delphi process involves an iterative process of multiple rounds, including generation of long lists (selected from existing items and new items added where required), feedback and voting.[3–6] Modified Delphi methodology includes at least 2 rounds of electronic questionnaires followed by a final round-table discussion and ratification round.[5] This study follows the modified Delphi approach (2 rounds of electronic questionnaires and a round table discussion). The Delphi study was conducted by an Executive Committee (PS, SJ and BC) who conceived, designed and executed the study. Participants of the Delphi study included a panel of 16 expert stakeholders (including the executive committee) from the fields of: obstetrics (1), maternal fetal medicine (2), postpartum recovery (3), pain (1), epidemiology (1), psychiatry (1), sleep (2), nursing and midwifery (1), PROM development (1) and patient representatives with lived experience of childbirth within the previous 5 years (3). The areas of expertise and race / ethnicity of Delphi participants is provided in eTable 1b.

The aim of the Delphi consensus was to develop a new PROM to measure well-being and health status, through the assessment of key domains (aspects/dimensions) of postpartum recovery, primarily for use at 6 weeks following childbirth, but also for use at different time points following hospital discharge up to 3 months postpartum.

Round 1 (January 2021): Questions from the outpatient recovery PROMs identified from the review of outpatient recovery measures were screened by stakeholders in order to determine the most applicable / appropriate items to assess relevant recovery domains (as previously proposed[7]) up to 12 weeks postpartum, following all delivery types. Questions were assigned to domains by the PROM experts within the group (PS and SJ). Stakeholders were invited to label each item (PROM survey question) as either ‘include’, ‘revise and reconsider’ or ‘exclude. Items

© 2025 Sultan P et al. *JAMA Network Open.*

selected as ‘include’ by ≥50% of participants proceeded to Round 2. All suggested amended versions of items, and newly proposed items also proceeded to Round 2 for further stakeholder evaluation.

Round 2 (February 2021): Stakeholders were invited to either ‘include,’ ‘revise and reconsider’ or ‘exclude’ items for Round 2. The proportions of selections to include from Round 1 were fed back to all stakeholders for their information and were not used to determine which questions would proceed to Round 3. Items proceeded to Round 3 if selected as ‘include’ by ≥70% of stakeholders or if a new suggested item was provided in Round 2. Consensus was achieved among the Executive Committee to utilize the cut-off value of 70% following review of the COMET (Core Outcome Measures in Effectiveness Trials) Handbook,[8] acknowledging that no broad agreement exists regarding what determines consensus.[9] This approach is also supported by a previously published study evaluating a long list of outcomes as was used in this study.[10]

Round 3 (April 2021): A recorded virtual round table discussion held using Zoom Video Communications software (San Jose, California), was attended by 13 out of the 16 stakeholders. Stakeholders were invited to discuss and vote to either include or exclude each individual question. The session was chaired by an Executive Committee member (PS) and co-chaired by another (BC). Comments and percentages of selections for inclusion and exclusion from Rounds 1 and 2 were disclosed to all stakeholders prior to the meeting. Questions which met the criteria for inclusion in Round 3, were discussed among the available stakeholders. Discussion was limited to 5-minutes per question and guided by the following factors: importance to postpartum recovery, importance to a specific recovery domain and ease of understanding of the question. Following discussion, stakeholders were invited to participate in live anonymized online polling, with stakeholders voting to either ‘include’ or ‘exclude’ each outcome. Using an iterative process, areas that warranted revision were modified and subsequent voting was undertaken. Digital recordings of the Round 3 discussions were sent to the 3 stakeholders who completed Rounds 1 and 2, but could not attend Round 3. All stakeholders were asked to approve the questions prior to cognitive debriefing interviews with postpartum individuals and the planned validation study.

The possible outcomes from this process were: (i) if ≥ 70% of stakeholders selected ‘include’ for a question, it was accepted; (ii) if < 70% selected ‘include’ then the question was excluded. Finally, a group consensus was reached regarding the final list of questions to include in the

© 2025 Sultan P et al. *JAMA Network Open.*

new STORK PROM. The wording of each item was kept in its original form or as close to, wherever possible, but alterations were made as required, in order for the grammar to be consistent with recommended PROMIS Likert response options based on expert advice (SJ).[11] Once the PROM questions for each domain were agreed upon by all study authors, the new PROM was then piloted on 10 postpartum individuals during a series of cognitive debriefing interviews as outlined below.

Statistical analysis
16 stakeholders was deemed to be an acceptable number of stakeholders in this Delphi study, since the interquartile range and range of participants in Delphi studies have previously been reported as [11-31] and 3 to 418, respectively.[9] Data were reported descriptively. Spreadsheets were developed for each round and circulated in Microsoft Excel (Excel for Mac; version 16.49, 2021) spreadsheet format. All denominator values for percentages were based on responses, and percentage values reported signify the proportion of stakeholders in agreement with a particular option.

Phase 3. Cognitive debriefing interviews: Recorded interviews were performed by a trained research assistant (PP, trained by SJ) with 10 postpartum adults who delivered a live neonate from any delivery mode at Stanford, and were able to speak and read English.[12] Interviews were performed until theoretical saturation was achieved, i.e. when no new concepts or ideas were discussed in 3 consecutive patient interviews. The purpose of the interviews was to obtain detailed end-user feedback regarding the newly developed STORK PROM to determine its interpretability, understanding, relevance to patient experience, and inconsistency in the impression of the proposed PROM questions selected in Round 3. Women were specifically asked about: a) comprehension of each question (the intent of the question, the meaning of terms used); b) memory retrieval of relevant information (what information do individuals need to recall and what types of strategies are used to retrieve information in order to answer the question?); c) verbal probing as previously described[12]; d) verbalization of thoughts when presented with each question and the basis of their response; e) decision process (was it answered accurately or was the answer given because it was expected of them?); f) response process (was the patient able to choose an appropriate answer?); g) general comments such as whether the PROM was too long; h) ease of understanding; i) interpretability; j) appropriateness of language; k) Likert scale and ease of use; l) should the question be reworded?

© 2025 Sultan P et al. *JAMA Network Open.*

Suggestions and responses made by the individuals during this phase were reviewed by the stakeholders and no further amendments were made to questions or the survey structure based on the interview findings. Readability of the finalized version of the STORK PROM was graded as: 1) Flesch Reading Ease score 63.8 (≥60 is good); 2) Flesch-Kincaid Grade Level 6.9 (≤8th grade level is desirable; Microsoft Word V16.82, 2022, Washington USA). The STORK PROM was then prepared for clinical validation in the phase 3 multicenter study as outlined below.

© 2025 Sultan P et al. *JAMA Network Open.*

eReferences 2.

1. Sultan P, Sharawi N, Blake L, et al. Use of patient-reported outcome measures to assess outpatient postpartum recovery: A systematic review. *JAMA Network Open*. 2021;4(5):e2111600.
2. *International Consortium for Health Outcomes Measurement (ICHOM). Pregnancy and Childbirth*. Accessed January 10, 2025. https://www.ichom.org/wp-content/uploads/2024/01/27-Pregnancy-Childbirth-Flyer.pdf
3. Basson R, Berman J, Burnett A, et al. Report of the international consensus development conference on female sexual dysfunction: definitions and classifications. *J Urol*. 2000;163(3):888-893.
4. Beattie E, Mackway-Jones K. A Delphi study to identify performance indicators for emergency medicine. *Emerg Med J*. 2004;21(1):47-50.
5. Diamond I, Grant R, Feldman B, et al. Defining consensus: A systematic review recommends methodologic criteria for reporting of Delphi studies. *J Clin Epidemiol*. 2014;67(4):401-409.
6. Eubank B, Mohtadi N, Lafave M, et al. Using the modified Delphi method to establish clinical consensus for the diagnosis and treatment of patients with rotator cuff pathology. *BMC Med Res Methodol*. 2016;20(16):56.
7. Sultan P, Jensen S, Taylor J, et al. Proposed domains for assessing postpartum recovery: A concept elicitation study. *British Journal of Obstetrics and Gynaecology*. 2022;129(1):9-20. doi:10.1111/1471-0528.16937
8. Williamson PR, Altman DG, Bagley H, et al. The COMET Handbook: Version 1.0. *Trials*. 2017;18(Suppl 3):1-50. doi:10.1186/s13063-017-1978-4
9. Boulkedid R, Abdoul H, Loustau M, Sibony O, Alberti C. Using and reporting the Delphi method for selecting healthcare quality indicators: A systematic review. *PLoS One*. 2011;6:e20476.
10. Blazeby J, Macefield R, Blencowe N, et al. Research Group of the Core Outcomes and iNformation SEts iN SUrgical Studies-Oesophageal Cancer; Consensus Group of the Core Outcomes and iNformation SEts iN SUrgical Studies-Oesophageal Cancer. Core information set for oesophageal cancer surgery. *Br J Surg*. 2015;102(8):936-943.
11. PROMIS. PROMIS® Instrument Development and Validation Scientific Standards Version 2.0.
12. Willis G. *Cognitive Interviewing: A Tool for Improving Questionnaire Design.* Sage; 2005.

© 2025 Sultan P et al. *JAMA Network Open.*

eMethods 2. Exploratory Factor Analysis of the STORK Questionnaire

Participants

Exploratory Factor Analysis (EFA) was conducted. Pooling observations across time can lead to some problems(1), such as multilevel correlations. observations at each time point were not large (i.e., the best sample size depended on various factors(2), however, there has been literature-wide discussion ranging from 100 to more than 1,000 observations indicating an absence of consensus regarding sample size(1, 3-6). Simulation studies conclude that EFA is a more appropriate procedure for large samples(5, 7) and compared to continuous variables, ordinal data require more samples(8)). It was possible to reach a robust factor solution by comparing the results of samples. Therefore, we included observations from postoperative week six (n=314).

Analysis

By applying EFA to emerge the latent structure of STORK questionnaire, we determined the appropriate number of factors and identified the items that are indicators of latent variables (i.e., factors)(9). Statistical analyses have been implemented in SPSS (26 version). A separate process of EFA was undertaken for each sample, including screening the data, evaluating the appropriateness of the EFA, determining elements of the EFA, and conducting the EFA to reach a factor solution. As a final step, a factor solution of each category was compared to determine the best factor solution.

Data screening involved inspection of both statistics and graphics(2, 10). Linearity, data distributions, outliers (univariate and multivariate), normality (univariate and multivariate), missing data, and reliability index were explored in this phase. Pearson correlation two-tailed test and scatterplots were used to assess linearity. Significant Pearson correlation among two pairs of items indicated there is a linearity correlation, however, for those that were non-significant, we used scatterplots. The minimum and maximum values of items were considered to detect univariate outliers. Mahalanobis distance (D^2) value and chi-square test based on Bonferroni adjustment were used to find multivariate outliers. The values of skew and kurtosis were used to assess univariate normality, which was deemed acceptable if univariate skew and kurtosis did not exceed 2.0 and 7.0, respectively(11). Mardia's multivariate normality tests(12) was done via Webpower application (https://webpower.psychstat.org/models/kurtosis/).

The pattern and frequencies of missing data were examined by items, observations, and all values. Any method of deletion or imputation will be accepted if less than 5–10% of the data are missing in a random pattern(13, 14). It is recommended to use complex multivariate techniques, such as multiple imputations, when more than 10% of the data are missing(15). The SPSS program used mean imputation method to impute missing data. As a measure of reliability, Cronbach's alpha was calculated.

We evaluated the appropriateness of the EFA using Bartlett's test of sphericity(16), Kaiser–Meyer–Olkin (KMO) measure of sampling adequacy(17) for both overall and each item, and singularity or multicollinearity index. KMO values less than 0.5 were not in the acceptable range(18). A correlation matrix's determinant was used to check for singularities or multicollinearity. A determinant greater than 0.00001 suggests there is no problem with multicollinearity(2).

We conducted the EFA based on the common factor model. Since ordinal data is not usually normally distributed and even categorization of continuous data leads to imprecise Pearson correlation(2). Methodologists have proposed that Pearson correlation are appropriate if the ordinal variables are measured by at least five categories(19). There were 47 ordinal items in the STORK questionnaire with 5 response categories. Factor extraction methods were Principal Axis Factoring (PAF)(5). We performed Parallel analysis to determine the number of factors(6). We used the following website to produce random eigenvalues since SPSS does not support this analysis; https://analytics.gonzaga.edu/parallelengine/. Rotations were chosen due to their simple

© 2025 Sultan P et al. *JAMA Network Open.*

structure and correlation between factors using Promax(2). It was recommended that a salient pattern coefficient of at least 0.32 was included as a threshold that was both practical and statistically significant(20, 21).

Root mean squared residual (RMSR) (<0.08 acceptable) was considered(22) as an index of model fit to evaluate the extracted factors. As an acceptable factor, at least three salient pattern coefficients, internal consistency reliability of ≥0.70, ideal values of model fit indices, and conceptually meaningful dimensions were considered(23). Sensitivity analyses were performed by assessing different categories of observations (POW 6 observations vs. full observations).

Results

1. Postpartum Week 6 (n=314)

1.1. Data screening

The items indicated no evidence of non-linearity using the Pearson correlation two-tailed test and scatterplots. The minimum and maximum values of all items were in a defined range, indicating no illegal or out-of-bounds values (i.e., no univariate outliers). Results revealed that the largest Mahalanobis distance (D^2) value was 179.16. However, 21 of the values using D^2 and chi-square distribution table were significant at 0.00016 (0.05/314) based on the Bonferroni adjustment.

Exploring univariate skew and kurtosis indicated 11 items exhibited high skew (q3 (-3.7), q4 (-2.3), q20 (-2.3), q22 (-3.1), q23 (-3.2), q28 (-8.2), q30 (-4.05), q33 (-3.1), q38 (-2.02), q40 (-2.8), and q46 (-3.03)). eight items indicated high kurtosis (q3 (15.17), q22 (10.1), q23 (11.8), q28 (75.2), q30 (19.3), q33 (12.2), q40 (8.5), and q46 (10.8)). These values suggested univariate nonnormality. Analysis of the Mardia's multivariate asymmetry skewness and kurtosis test using the Webpower application indicated that the data were not multivariate normal (multivariate kurtosis; 2865.3, (p<0.001) and multivariate skewness; 829.86 (p<0.001)).

As displayed in the following figure, 27 variables from 47 variables (items) included missing data (57.45%). However, 281 cases (observations) were complete and only 3 (10.51%) contained missing data. With 314 cases and 47 variables, there were 14758 data cells. Of those, 14719 were complete and 39 were missing (0.26%). This trivial amount of missing data can probably be ignored. Due to less than 10% percent of data being missing considering all data cells, we used mean imputation for conducting EFA.

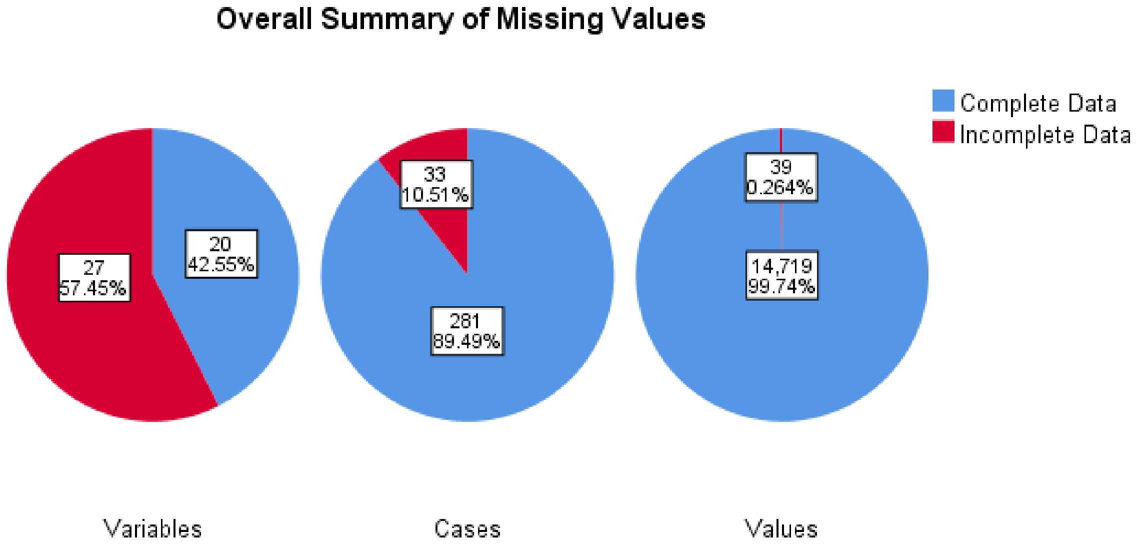

© 2025 Sultan P et al. *JAMA Network Open.*

The internal reliability of STORK questionnaire was investigated using Cronbach's alpha. Results indicated that the alpha for the total scale was equal to 0.930. There was no item with zero variance.

1.2. Appropriateness of the EFA

A visual scan of the correlation matrix for the data revealed that most of the coefficients were ≥ 0.30, but none exceeded 0.09. Bartlett's test of sphericity was statistically significant (p <.001). The KMO measure of sampling adequacy was acceptable, with values of 0.892 for the total model and 0.728(q40) to 0.942(q27) for each of the measured variables.
The determinant of the correlation matrix was 0.000000000002423(2.423e-12) which was lower than 0.00001. Therefore, singularity or multicollinearity was the issue of this sample.

EFA

1.3.1. SPSS outputs

Based on Parallel analysis, three factors were selected to run exploratory factor analysis. It was based on Pearson correlation, Principal axis factoring, and Promax rotation. We considered a coefficient of more than 0.32 in the final factor solution.

N	Item statement	Factor			
		1	2	3	4
1	I have been able to perform my usual activities	.401	-.024	.304	.048
2	How satisfied have you been with your ability to perform your daily living activities?	.378	-.024	.348	.119
3	I was able to stand up from sitting down	.593	-.147	-.066	.073
4	I was able to bend, kneel or stoop	.647	-.111	.040	.052
5	How satisfied have you been with your vaginal or cesarean healing following delivery?	.530	-.185	.141	.095
6	I have been interested in sexual activities	.105	-.163	.309	.065
7	How much has your physical health been affected by your recent childbirth?	.527	-.084	.172	.012
8	Did you have difficulty performing work or other activities (for example, it took extra effort) as a result of your physical health	.768	-.109	.043	.030
9	To what degree did fatigue interfere with your physical functioning?	.287	.056	.533	-.049
10	How much have you needed medical treatment (medicines or healthcare professional input) to function in your daily life?	.686	.050	-.138	-.001
11	How much did pain interfere with your day-to-day activities?	.887	-.029	-.074	-.080
12	I experienced pelvic pain	.612	.186	-.056	-.212
13	I was concerned about breast soreness	.158	.164	.159	.051
14	I was satisfied with my decisions about my baby's health and well being	-.047	.063	-.003	.539
15	I have been satisfied with the way my body looks	.073	-.025	.378	.068
16	I have been bothered by aches or pain	.737	-.015	.009	-.046
17	How much has pain interfered with your mood?	.745	.107	.043	.020

© 2025 Sultan P et al. *JAMA Network Open.*

18	How much did pain interfere with your enjoyment of life?	.803	.066	-.036	.027
19	My worries overwhelmed me	.046	.684	.041	.153
20	I felt helpless	-.013	.752	-.074	.097
21	I felt anxious	-.074	.650	.198	.083
22	I felt worthless	-.086	.881	-.073	-.068
23	I felt hopeless	-.035	.815	-.118	.018
24	I felt lonely	-.078	.707	.155	-.076
25	How much conflict have you felt between childcare and work?	.155	.137	.162	.055
26	How much difficulty did you have remembering the important things?	.116	.424	.235	.000
27	How much difficulty did you have problem-solving in day-to-day life?	.202	.451	.129	.045
28	I have had thoughts that life is not worth living	.033	.457	-.179	-.034
29	How satisfied are you with your access to medical care?	.253	-.066	-.072	.394
30	How safe do you feel in your daily life?	.103	.016	-.108	.370
31	How satisfied are you with your ability to feed your new baby?	.037	-.105	.001	.602
32	I am comfortable with how I have fed my baby (either bottle, breast or pump)	-.049	-.134	.073	.715
33	I have been taking good care of my baby's physical needs (feedings, changing diapers, doctor's appointments)	.021	.018	-.051	.524
34	I trusted my own feelings (instincts) when it came to taking care of my baby	-.014	-.035	-.062	.670
35	I was able to relax and enjoy time with my baby	-.064	.068	.266	.411
36	How satisfied have you been with your baby's health?	-.072	-.019	.116	.668
37	I have had concerns about my infant's growth and development	.012	.171	-.170	.547
38	Anxiety or worry interfered with my mothering ability	-.059	.501	.023	.331
39	Do you worry about your finances?	.040	.428	.155	-.042
40	Do you worry about an unexpected pregnancy?	-.061	.527	-.084	-.139
41	How satisfied have you been with your sleep?	-.080	-.133	.808	.081
42	I got enough sleep to feel rested in the morning	-.149	-.097	.985	-.045
43	Did you have enough time to rest?	-.120	-.067	.894	-.012
44	I had difficulty falling or staying asleep	-.072	.257	.371	-.108
45	I felt sleepy during the daytime	.060	.098	.717	-.153
46	How much has pain interfered with your sleep?	.560	.270	-.209	.012
47	I felt fatigued	.034	.113	.707	-.075

By considering the strongest loading value (dark green cells) for each item, the model factors were as follows:

- Factor1: q1-q5, q7-q8, q10-q12, q16-18, q46 (14 items) (physical function and pain)
- Factor2: q19-q24, q26-q28, q40, q38-q39 (12 items) (mental function)
- Factor3: q9, q15, q41-q45, q47 (8 items) (fatigue, sleep, body looks)

© 2025 Sultan P et al. *JAMA Network Open.*

- Factor4: q14, q29-q30, q31-q37 (10 items) (baby, feel safe, access to medical care)

Items with an inadequate coefficient (>0.32), including q6, q13, and q25 were removed (3 items).

1.3.2. Reliability of factors

The factors exhibited alpha reliability coefficients of 0.91, 95% CI [0.89, 0.92] for factor 1 (14 items), 0.88, 95% CI [0.86, 0.90] for the value of factor 2 (12 items), 0.86, 95% CI [0.83, 0.88] for the value of factor 3 (8 items), and 0.80, 95% CI [0.76, 0.83] for the value of factor 4 (10 items).

1.3.3. Average overall residual misfit

The RMSR for the four-factor model was 0.047, and there were 30 residual coefficients greater than 0.10, suggesting that a lot of residual variances remained after extracting four factors. Essentially, this model appeared to be relatively acceptable in terms of RMSR.

- RMSR = 0.047
    - >.05 = 249 (23.03%)
    - >.10 = 30 (2.78%)
    - >.30 = 0 (0.0%)
    - Largest = +0.289

1.3.4. Changing the number of factors

Increasing or decreasing the number of factors did not enhance factor matrix.

© 2025 Sultan P et al. *JAMA Network Open.*

eReferences 3.

1. Comrey AL, Lee HB. A first course in factor analysis: Psychology press; 2013.
2. Watkins MW. A step-by-step guide to exploratory factor analysis with SPSS: Routledge; 2021.
3. Schönbrodt FD, Perugini M. At what sample size do correlations stabilize? Journal of Research in Personality. 2013;47(5):609-12.
4. Child D. The essentials of factor analysis: A&C Black; 2006.
5. Gaskin CJ, Happell B. On exploratory factor analysis: A review of recent evidence, an assessment of current practice, and recommendations for future use. International journal of nursing studies. 2014;51(3):511-21.
6. Howard MC. A review of exploratory factor analysis decisions and overview of current practices: What we are doing and how can we improve? International journal of human-computer interaction. 2016;32(1):51-62.
7. Costello AB, Osborne J. Best practices in exploratory factor analysis: Four recommendations for getting the most from your analysis. Practical assessment, research, and evaluation. 2005;10(1):7.
8. Rouquette A, Falissard B. Sample size requirements for the internal validation of psychiatric scales. International journal of methods in psychiatric research. 2011;20(4):235-49.
9. Brown TA. Confirmatory factor analysis for applied research: Guilford publications; 2015.
10. Hoelzle JB, J. Meyer G. Exploratory factor analysis: Basics and beyond. Handbook of Psychology, Second Edition. 2012;2.
11. Curran PJ, West SG, Finch JF. The robustness of test statistics to nonnormality and specification error in confirmatory factor analysis. Psychological methods. 1996;1(1):16.
12. Mardia KV. Measures of multivariate skewness and kurtosis with applications. Biometrika. 1970;57(3):519-30.
13. Chen S-F, Wang S, Chen C-Y. A simulation study using EFA and CFA programs based the impact of missing data on test dimensionality. Expert Systems with Applications. 2012;39(4):4026-31.
14. Tabachnick BG, Fidell LS, Ullman JB. Using multivariate statistics: pearson Boston, MA; 2013.
15. Newman DA. Missing data: Five practical guidelines. Organizational Research Methods. 2014;17(4):372-411.
16. Bartlett MS. Tests of significance in factor analysis. British journal of psychology. 1950.
17. Kaiser HF. An index of factorial simplicity. psychometrika. 1974;39(1):31-6.
18. Dziuban CD, Shirkey EC. When is a correlation matrix appropriate for factor analysis? Some decision rules. Psychological bulletin. 1974;81(6):358.
19. Rogers P. Best practices for your exploratory factor analysis: A factor tutorial. Revista de Administração Contemporânea. 2022;26.
20. Bandalos DL, Gerstner JJ. Using factor analysis in test construction. 2016.
21. Norman G, Streiner D. Logistic and Poisson regression. Biostatistics: the bare essentials 4th ed Shelton, CT: People's Medical Publishing House. 2014:169-80.
22. Finch WH. Using fit statistic differences to determine the optimal number of factors to retain in an exploratory factor analysis. Educational and psychological measurement. 2020;80(2):217-41.
23. Watkins MW. Exploratory factor analysis: A guide to best practice. Journal of Black Psychology. 2018;44(3):219-46.

© 2025 Sultan P et al. *JAMA Network Open.*
