# Supplementary material for: Development and Validation of the Stanford Obstetric Recovery Checklist (STORK): A Delphi Consensus and Multicenter Clinical Validation Study
Source: JAMA Netw Open. 2025 Apr 17;8(4):e255713. doi: 10.1001/jamanetworkopen.2025.5713 (PMC12006865; doi:10.1001/jamanetworkopen.2025.5713)
Supplement: Supplement 2. — Nonauthor Collaborators. Stanford Obstetric Recovery Checklist (STORK) Investigators [file jamanetwopen-e255713-s002.pdf]

Supplemental Online Content: Nonauthor Collaborators
*First name, last name, and suffix (if applicable) are required and will appear in PubMed.

***Group Name(s): Stanford Obstetric Recovery Checklist (STORK) Investigators**

***First Name and Middle Initial(s)**	***Last Name**	***Suffix (eg, Jr, III)**	Academic Degrees	Institution	Location (city, state/province, country)	Role or Contribution, eg, chair, principal investigator	Group (if more than 1 Group listed in the byline) and/or Subgroup (eg, Steering
Jessica R	Ansari	Dr.	Medical Degree	Stanford University	CA	Patient Stakeholder: provided intellectual content and helped with developmnet of survey instrument	
Fiona	Barwick	Dr.	Medical Degree	Stanford University	CA	Expert Stakeholder: sleep medicine specialist. Provided intellectual content and helped with developmnet of survey instrument	
Kathleen F	Brookfiled	Dr.	Medical Degree	University of Arkansas for Medical Sciences	AR	Expert Stakeholder: sleep medicine specialist. Provided intellectual content and helped with developmnet of survey instrument	
Suzan	Carmichael	Dr	PhD	Stanford University	CA	Expert Stakeholder: epidemiologist. Provided intellectual content and helped with developmnet of survey instrument	
Jessica	Coker	Dr	Medical Degree	University of Arkansas for Medical Sciences	AR	Expert Stakeholder: psychiatrist. Provided intellectual content and helped with developmnet of survey instrument	
Yasser Y	El Sayed	Dr.	Medical Degree	Stanford University	CA	Involved in study conception and study design. Obstetrician and MFM	
Pamela	Flood	Dr	Medical Degree	Stanford University	CA	Expert Stakeholder: pain specialist. Provided intellectual content and helped with developmnet of survey instrument	
Cedar	Fowler	Dr	Medical Degree	Stanford University	CA	Involved in preparation of survey instruments using REDCap electronic software	
Makoto	Kawai	Dr.	Medical Degree	Stanford University	CA	Expert Stakeholder: sleep medicine specialist. Provided intellectual content and helped with developmnet of survey instrument	
Colleen	Moreno	Dr.	Medical Degree	Stanford University	CA	Expert Stakeholder: director of midwifery and nursing. Provided intellectual content and helped with developmnet of survey instrument	
James	O'Carroll	Dr.	Medical Degree	Stanford University	CA	Involved in multicenter study including IRB approval and preparation of study documents	
Nadir	Sharawi	Dr.	Medical Degree	University of Arkansas for Medical Sciences	AR	Expert Stakeholder: postpartum recovery specialist. Provided intellectual content and helped with developmnet of survey instrument	
Ellile	Sultan	Dr.	Medical Degree		AR	Patient Stakeholder: provided intellectual content and helped with developmnet of survey instrument	
Julie	Whittington	Dr	Medical Degree	Naval medical center portsmouth, VA	VA	Expert Stakeholder: OB / MFM specialist. Provided intellectual content and helped with developmnet of survey instrument	
Romy	Yun	Dr	Medical Degree	Stanford University	CA	Patient Stakeholder: provided intellectual content and helped with developmnet of survey instrument	
