# Supplementary material for: Development and Validation of the Stanford Obstetric Recovery Checklist (STORK): A Delphi Consensus and Multicenter Clinical Validation Study
Source: JAMA Netw Open. 2025 Apr 17;8(4):e255713. doi: 10.1001/jamanetworkopen.2025.5713 (PMC12006865; doi:10.1001/jamanetworkopen.2025.5713)
Supplement: Supplement 3. — Data Sharing Statement [file jamanetwopen-e255713-s003.pdf]

Data Sharing Statement

Sultan. Development and Validation of the Stanford Obstetric Recovery Checklist (STORK). *JAMA Netw Open*. Published April 17, 2025. doi:10.1001/jamanetworkopen.2025.5713

Data
Data available: Yes
Data types: Other (please specify)
Additional Information: de-identified data will be made available upon reasonable request
How to access data: psultan@stanford.edu
When available: With publication

Supporting Documents
Document types: None

Additional Information
Who can access the data: upon reasonable request
Types of analyses: upon reasonable request
Mechanisms of data availability: with investigator support
